# Gastrointestinal Hemorrhage in Acute Kidney Injury Patients on Hemodialysis

**DOI:** 10.7759/cureus.5652

**Published:** 2019-09-13

**Authors:** Shantanu Solanki, Khwaja F Haq, Muhammad Ali Khan, Raja Chandra Chakinala, Siddharth Mehta, Khwaja S Haq, Uvesh Mansuri, Zubair Khan, Darshan Gandhi, Jagmeet Singh, Savneek S Chugh

**Affiliations:** 1 Hospital-based Medicine, Guthrie Robert Packer Hospital, Sayre, USA; 2 Gastroenterology, Henry Ford Hospital, Detroit, USA; 3 Medicine, University of Tennessee Health Science Center, Memphis, USA; 4 Internal Medicine, Guthrie Robert Packer Hospital, Sayre, USA; 5 Medicine, Trumbull Regional Medical Center, Youngstown, USA; 6 Medicine, Kingsbrook Jewish Medical Center, Brooklyn, USA; 7 Medicine, Medstar Union Memorial Hospital, Baltimore, USA; 8 Internal Medicine, University of Toledo Medical Center, Toledo, USA; 9 Radiology, St. Vincent’s Medical Center, Bridgeport, USA; 10 Nephrology, Guthrie Robert Packer Hospital, Sayre, USA; 11 Medicine/Nephrology, New York Medical College at Westchester Medical Center, Valhalla, USA

**Keywords:** gastrointestinal bleed (gib), acute kidney injury (aki), hemodialysis (hd)

## Abstract

Background

Gastrointestinal bleeding (GIB) has been reported to be more common in patients with chronic renal failure and end-stage renal disease requiring hemodialysis with higher mortality than in the general population. Limited epidemiological data exist on the annual number of hospitalizations, demographic variation, cost of care, and outcomes for GIB in patients with acute kidney injury (AKI) requiring and not requiring hemodialysis (HD). The main objective of this study was to analyze the trends of GIB in patients with AKI requiring HD and those not requiring HD during hospitalization.

Methods and Results

We analyzed the National (Nationwide) Inpatient Sample (NIS) database for all subjects with a discharge diagnosis of AKI as the primary or secondary diagnosis during the period from 2001 to 2011. Subjects with a discharge diagnosis of hemodialysis and GIB were then identified from the pool and trends were analyzed. A significant rise in the annual number of hospitalizations with AKI was found with a greater proportion being discharged without HD. From 2001 to 2011, there were 19,393,811 hospitalizations with a discharge diagnosis of AKI of which 1,424,692 (7.3%) received HD (HD group), whereas 17,969,119 (92.7%) did not receive HD (non-HD group) (p < 0.0001). The male gender was more commonly affected by GIB than the female gender in both groups (p < 0.0001). The cost of care per hospitalization for GIB patients in the HD group increased over the study period with average found to be $61,463 (adjusted for inflation, p < 0.0001), whereas for GIB patients in the non-HD group, it showed a slight decrease in trend with the average found to be $28,419 (p < 0.0001). All-cause mortality was higher for GIB patients in the HD group (38.1%) than in the non-HD group (25.1%) (p < 0.0001).

Conclusions

GIB is more common and associated with higher all-cause inpatient mortality in patients receiving HD in comparison to non-HD patients.

## Introduction

Gastrointestinal bleeding (GIB) is more common in acute kidney injury (AKI) patients requiring hemodialysis than in the general population. However, the estimated burden of inpatient treatment of GIB in AKI in the United States (US) is still unknown. Hence, an accurate understanding of the trends in AKI-related hospitalizations leading to GIB is necessary for appropriate healthcare planning. Also, a national study is more likely to identify vulnerable groups and reduce disparities in healthcare than a single center and/or multicenter studies [1-2]. Thus, we designed our study specifically to identify such groups.

## Materials and methods

Source of data

The National (Nationwide) Inpatient Sample (NIS), designed by the Agency for Healthcare Research and Quality (AHRQ), is the largest all-payer inpatient database in the US. This data is compiled yearly and contains discharge data from over 1,200 hospitals located across 45 states in the US. The NIS was designed to approximate a 20% stratified sample of community hospitals in the country and provides sampling weights to calculate national estimates [3]. The NIS contains information included in a typical discharge summary, with safeguards in place to protect the privacy of individual patients, physicians, and hospitals. Each individual hospitalization is de-identified and maintained in the NIS as a unique entry with one primary discharge diagnosis and up to 24 secondary diagnoses during that hospitalization. Each entry also carries information on demographic details, insurance status, comorbidities, primary/secondary procedures, hospitalization outcomes, length of stay, and cost of care. The internal validity of the database is guaranteed by annual data quality assessments of the sample. Moreover, comparisons with data sources, such as the American Hospital Association (AHA) Annual Survey of Hospitals, National Hospital Discharge Survey from the National Center for Health Statistics, and MedPAR inpatient data from the Centers for Medicare and Medicaid Services, strengthen the external validity of the sample [4-5].

Study design

We queried the NIS database from the years 2001 to 2011 to conduct a cross-sectional study to identify all hospitalizations with AKI. We extracted data regarding all hospitalizations from 2001 to 2011 with a primary or secondary diagnosis of AKI, which were identified with validated International Classification of Diseases, 9th Revision (ICD-9), Clinical Modification: ICD-9 codes 584.x, 634.3, 635.3, 636.3, 637.3, 638.3, 639.3, 669.3, and 958.5. Hospitalizations with a discharge diagnosis of hemodialysis (HD) (ICD-9 code 39.95) and GIB (ICD-9 code 578.x) were then identified from the pool. Patients less than 18 years of age were excluded. Also, all hospitalizations with information missing about age, sex, admission/discharge date, and in-hospital mortality status were excluded to gather and document accurate demographics of AKI hospitalizations as seen in previous national studies [2, 6]. To calculate the estimated cost of hospitalizations, the NIS data was merged with cost-to-charge ratios (CCR) files available from the Healthcare Cost and Utilization Project. We estimated the cost of each inpatient stay by multiplying the total hospital charge with the cost-to-charge ratio. 

Variables and statistical analysis

SAS 9.4 (SAS Institute Inc., Cary, NC, USA) was utilized for statistical analyses. Since the NIS represents a 20% stratified random sample of US hospitals, analyses were performed using hospital-level discharge weights, provided by the NIS, to obtain national estimates of hospitalizations [3]. AKI-related hospitalizations per million US population were calculated by dividing the number of such hospitalizations in each year by the US census population greater than 18 years of age for that year. AKI hospitalizations were also calculated in subgroups of age (18 - 34, 35 - 49, 50 - 64, 65 - 79, and > 80 years), sex, race (White, Black, Hispanic, and Others), insurance status (Medicare/Medicaid, private insurance, self-pay/other), hospital region (Northeast, Midwest, South, West), and teaching status of the hospital. According to the AHRQ, a hospital is considered to be a teaching hospital if it: 1) has an Americal Medical Association (AMA)-approved residency program; 2) is a member of the Council of Teaching Hospitals; or 3) is a hospital with a full-time equivalent ratio of interns and/or residents to beds of more than 0.25 [7]. For categorical variables (e.g., the annual change in AKI hospitalization rate and in-hospital mortality), trends were assessed using the Cochrane Armitage Trend Test [8]. For continuous variables (e.g., cost of hospitalization), a non-parametric test for trend (the Wilcoxon rank-sum test) was used [9].

## Results

A total of 19,393,811 hospitalizations for AKI (as the primary or secondary discharge diagnosis) were identified in the US population from 2001 to 2011 (Table 1). AKI hospitalizations progressively increased from 685,656 in 2001 to 3,179,489 in 2011 (p < 0.0001). There were 1,424,692 (7.3%) hospitalizations in the HD group and 17,969,119 (92.7%) hospitalizations in the non-HD group. The total number of discharges in both groups showed a significant rise over the study period (p < 0.0001).

**Table 1:** Total Number of AKI Hospitalizations - With HD vs. Without HD AKI - acute kidney injury; HD - hemodialysis

	Total hospitalizations with AKI	HD Group	Non-HD Group
2001	685,656	75,407	610,249
2002	820,630	83,852	736,778
2003	974,643	95,674	878,970
2004	1,142,788	105,488	1,037,300
2005	1,320,133	119,048	1,201,085
2006	1,544,533	127,384	1,417,149
2007	1,853,080	137,701	1,715,379
2008	2,303,280	156,033	2,147,247
2009	2,659,757	171,372	2,488,385
2010	2,909,823	175,488	2,734,335
2011	3,179,489	177,246	3,002,243
Total	19,393,811	1,424,692	17,969,119

In both groups having GIB, patients were predominantly White (56.4% in the HD group vs 56.1 in the non-HD group) and between 65 to 79 years of age (34.7% in Group I vs 34.0% in Group II). There were more hospitalizations in males in both groups (53.2% in the HD group vs. 52.4% in the non-HD group). GIB was reported in 5.3% of the HD group discharges as compared to 3.5% of the non-HD group discharges (p < 0.0001). All-cause mortality during the hospital stay was found to be 38.1% for Group 1 and 25.1% for Group II. The average cost of care for hospitalization with GIB in the HD group was higher than in the non-HD group ($61,463 vs. $28,419, respectively; p < 0.0001).

## Discussion

Based on our literature review, we found that this is the first study which looks at the association of hemodialysis with gastrointestinal hemorrhage in the US population. We found that hospitalized patients on dialysis have a higher risk of gastrointestinal bleeding and mortality than those who do not require dialysis. These findings are similar to those found in a study from Taiwan reported in 2015 [10].

Luo and colleagues also found that patients with end-stage renal disease undergoing hemodialysis had a high risk of ulcer bleeding [11]. In the US, upper GIB events reported in an outpatient setting in the Medicare population was 40% according to one study [12].

The mechanisms leading to a higher incidence of GIB in such patient populations are still unknown. The suggested mechanisms are as follows:

1. Patients receiving hemodialysis are frequently exposed to anticoagulants, which augment the bleeding risk. However, the presence of end-stage renal disease (ESRD) itself might mask the effects of anticoagulation. Thus, it is hard to prove a direct causal relationship [3, 13].

2. A higher incidence of gastric and duodenal ulcers has been seen in patients undergoing dialysis [14-15].

3. Chronic kidney disease itself can be an independent risk factor, causing hemodynamic instability in the gastrointestinal blood supply, and thus leading to impaired healing in patients with preexisting GI ulcers [11].

4. Platelet dysfunction, platelet-vessel wall interaction, and abnormalities in blood coagulation are plausible explanations [16].

Several studies have reported independent risk factors responsible for GIB in patients with kidney disease. Wasse and colleagues reported that cardiovascular disease, smoking, and physical inactivity independently were risk factors for bleeding in the upper gastrointestinal tract [16]. Chen and colleagues found that diabetes, congestive heart failure, and low albumin levels were risk factors for peptic ulcers in patients receiving dialysis [15]. We found that patients undergoing dialysis for AKI had a higher risk of GIB. Further studies are required to find the mechanisms leading to a higher incidence of GIB. 

Our study found that dialysis-requiring AKI was associated with a higher risk of upper and lower GIB. Also, bleeding was a significant predictor of all-cause mortality after undergoing HD. Our study reports important findings associated with AKI-related hospitalizations over an 11-year period in the US. The abstract for our study was presented at the National Kidney Foundation Spring Clinical Meeting 2017 [17]. The main findings of our analysis of the largest publicly available national database available are:

1) GIB is more common in HD patients than non-HD patients;

2) Mortality is higher in HD patients developing GIB when compared to non-HD patients;

3) The overall cost of hospitalization for GIB in HD patients is more than that in non-HD patients.

Limitations

Administrative databases are susceptible to errors arising from coding inaccuracies. AKI diagnosis and presence of comorbidities were based on the presence of administrative codes.

1) ICD-9 CM codes 584.x, 634.3, 635.3, 636.3, 637.3, 638.3, 639.3, 669.3, and 958.5 are validated for AKI (with and without bleeding, respectively) in the administrative database. However, this database does not allow us to identify new onset AKI.

2) Overestimation: The NIS considers each hospitalization as a separate entry so there is no coding method that can separate index cases from readmissions. This could result in an overestimation of the number of admissions.

3) The design of the database allowed us to examine in-hospital characteristics only; hence, the study does not provide insight into any long-term follow-up outcomes.

4) Our study focused on AKI-related inpatient hospitalizations and did not include any data pertaining to the care of patients undergoing hemodialysis in outpatient settings.

Strengths

Despite these limitations, our study has the following important strengths:

1) Large sample size; hence, the greater statistical power of the study;

2) The absence of selection bias associated with clinical trials;

3) Sample population representative of the entire nation and not confined to a group, hospital, race, or region.

## Conclusions

In conclusion, on reviewing the hospitalization trends over the last decade, we found a consistent significant increase in hospitalizations associated with GIB in renal failure patients, along with an increase in the length of stay and cost of care without any substantial change in mortality trends over the last decade. A reduction in GIB can probably be a favorable outcome of implementing preventative and prophylactic measures in renal failure patients undergoing hemodialysis. The cost-effectiveness of the current management of AKI and mortality benefits need further evaluation of the additional risk factors and identification of their trends to elucidate our findings. We believe that prevention of GIB in HD patients with measures including (but not limited to) early identification and effective outpatient management will further reduce the burden on the healthcare system.
